# Supplementary material for: Chromothripsis during telomere crisis is independent of NHEJ, and consistent with a replicative origin
Source: Genome Res. 2019 May;29(5):737–49. doi: 10.1101/gr.240705.118 (PMC6499312; doi:10.1101/gr.240705.118)
Supplement: Supplemental Material [file supp_gr.240705.118_Supplemental_file_1.zip › contigs/annotated_contigs/DB39/contig.3.DB39_length_341_mean_cov_44.4662756598.docx]

**DB39_length_341_mean_cov_44.4662756598**

```
A|AAGAAAAAAAACAAGAAAGAAAGCAAGAAAGCAAGAAAGCAAGCAAGAAAGAAACGAAAGAAAGAAAGAAAGCAAGAAA|ACG|GGA
  >chr7:116223550-116223628 + E=3e-13 p=0e+00                                       >c
AAGCAAGAAAGCACGAAAGCAAGCAAGC|GAGC|GAGAGAGAGAGAGAGAGAGAGAGAGGCGGGGCGCGGTGGCTCACGCGTGTCATCC
hr1:112713402-112713433 + E=3e-04  >chrUn_gl000220:102216-102439 - E=6e-117
CAGCACTTTGGGAGGCTAAGGCAGGCGGACCACCTGAGGTTGGGAGTGGGAGACCAGCCTGACCAACATGGAAAAACACCGTCTCTACT

AAAAGTACAAACATCAGCCAGGCACGGTGGCCCATGCCTGTAATCCCAGCTAATCAGGAGGCTGAGGCAGGAGAATCGC
```
